# Movement behaviours and adherence to guidelines: perceptions of a sample of UK parents with children 0–18 months

**DOI:** 10.1186/s12966-022-01300-5

**Published:** 2022-05-21

**Authors:** Kathryn R. Hesketh, Xanne Janssen

**Affiliations:** 1 MRC Epidemiology Unit, School of Clinical Medicine, University of Cambridge, Cambridge, UK; 2 Physical Activity for Health Group, School of Psychological Sciences and Health, University of Strathclyde, Glasgow, Scotland G1 1QN UK

**Keywords:** Tummy time, Physical activity, Sleep, Restraint, Infants, Sedentary behaviour

## Abstract

**Background:**

Movement behaviours are important for infant (0–12 mo) and toddler (1–2 yrs) health and development, yet very little is known about adherence to the 24-hour movement behaviour guidelines and parents perception of these behaviours in these age groups. This study aimed to examine parental perceptions of movement behaviours and adherence to guidelines in a sample of UK parents with children 0–18 months.

**Methods:**

Participants were 216 parent-child dyads from the cross-sectional Movement Behaviour Assessment in Infants and Toddlers (M-BAIT) study. Tummy time, screen time, restraint time and sleep were measured using a parental questionnaire. A sub-sample of parents were asked about their priority areas for their child’s health and development. Frequencies were used to describe the proportion of children meeting movement behaviour guidelines, the number of guidelines met and priority areas for parents. Mann-Whitney U-tests (continuous variables) and chi-square tests (categorical variables) were used to assess the differences between boys and girls.

**Results:**

For those under 12 months of age, just over 30% of children met tummy time recommendations, 41.3% met the screen time guidelines, 57.8% met restraint guidelines and 76.2% met sleep guidelines. For those 12 months and over, 24.1% met the screen time guidelines, 56.9% met restraint guidelines and 82.8% met sleep guidelines. Parents identified sleep and physical activity as top priorities for their child. Limiting screen time was deemed least important.

**Conclusion:**

In this sample of UK infants and toddlers (0–18 months), few adhered to the sedentary behaviour and tummy time guidelines, whereas the majority meet sleep guidelines. This mirrors parental priorities; limiting screen time was seen as less important, with sleep and physical activity deemed most important. These findings suggest greater efforts are needed to raise awareness about screen and tummy time, supporting parents and care-providers to promote positive movement behaviours.

**Supplementary Information:**

The online version contains supplementary material available at 10.1186/s12966-022-01300-5.

## Background

Sufficient physical activity and sleep, along with limited screen time is known to confer significant benefits to children’s physical and psychosocial health [1–3]. In preschool-aged children (3–5 years), engaging in appropriate levels of these behaviours has been associated with a decreased risk of adiposity, and improved motor skill development, psychosocial health, and cardiometabolic indicators [3–5]. With increased research in very young children (0–24 months), evidence is also starting to emerge of the benefits of movement behaviours for adiposity indicators, motor skills, cognitive development and bone and skeletal health in this age group [1, 3, 4]. For example, focussing on tummy time, infants who spend more time prone appear to have better gross motor and total development, reductions in their BMI-z scores, and lower risk of brachycephaly [6].

Given these health benefits, international guidelines now detail age-specific physical activity, sedentary behaviour and sleep recommendations for infants, toddlers and preschool-aged children (e.g [7–9]). Infants under 12 months old are recommended to engage in at least 30 minutes tummy time spread throughout the day, with those 12 months and over encouraged to participate in at least 180 minutes of physical activity each day. Screen time is not recommended for those under 2 years of age and the amount of time children spend restrained while awake should also be minimised to 1 hour at a time. Sleep recommendations range from 11 to 17 hours per 24 hours, depending on the age of the child [7–9].

Despite many countries issuing early years guidance, no country to date has an appropriate method to allow for national surveillance of physical activity, sleep and sedentary behaviour in infants and toddlers. This means there is relatively little evidence at present about levels of adherence to these guidelines in the under 2 s. Having valid and reliable methods to assess movement behaviours, and the time children spend therein, is therefore vitally important for this burgeoning field of research. Whilst activity monitors are commonly used in older children and adults, in younger children, it is difficult to disentangle child- from adult-initiated movements (e.g. whether a child is being picked up and carried or moving independently). In addition, movement in infants (< 12 mo, and more specifically in those <6mo) is largely achieved through limb/ head movement and rolling only. Studies that have assessed infants and toddlers adherence to the movement behaviour guidelines report low levels of adherence to all three guidelines, ranging from 3.5 to 19.1% [10].

Despite a perception that young children are naturally or innately active, care-givers, the child’s environment and cultural practices may inadvertently prevent children from moving as freely as they would like. For example, children now spend increasing amounts of time restrained in car seats and highchairs, limiting their ability to be active [11]. Parents’ behaviours, views and priorities play an important role in a child’s daily behaviour [10, 12]. Understanding parents’ priorities may therefore be key in increasing infants’ and toddlers’ physical activity and sleep, and reducing screen time. This study therefore sought to develop to the current limited evidence base in under 2 s, exploring parental perceptions of levels and adherence to movement behaviours and guidelines in a sample of UK parents with children 0–18 months.

## Methods

### Design and participants

This was a UK based cross-sectional study: participants were recruited as part of the Movement Behaviour Assessment in Infants and Toddlers (M-BAIT) study via social media in July 2020 and June 2021. Participants were eligible to participate if they were a UK resident, aged 18 years or older, and a parent or caregiver of a child aged 0–18 months old. Approval for the M-BAIT study was given by the School of Psychological Sciences and Health Ethics Committee at University of Strathclyde (A 62/20/05/2020/A).

### Measures

Participants were asked to complete an online questionnaire derived for the M-BAIT study. The questionnaire included questions about their child’s physical activity, sedentary behaviour and sleep, and areas parents identified as priorities for their child (e.g. sleep, diet, physical activity). Participants also reported their child’s and their own date of birth, sex, employment status and whether or not their child attended childcare.

#### Physical activity, sedentary behaviour and sleep

Questions assessing physical activity, sedentary behaviour and sleep were based on published questionnaires, previously used in infants and toddler [13, 14]. Although the sleep questionnaire has been validated previously [14], the validity of the physical activity and sedentary behaviour questionnaire is unknown [13]. Parents were asked to report the time a child spent being physically active with an adult, other child, or alone as well as time spent outside or playing on the floor over an average week. In addition, parents were asked to report duration of their child’s tummy time. Reported time spent in each behaviour was then divided by 7 to obtain average time per day. For those under 12 months, those reporting more than 30 min/day of tummy time were classed as meeting the physical activity guidelines.

Parents reported weekly time spent watching a screen, as well time spent in five situations in which the child would be restrained while awake (bouncer/swing, stroller/pram, car seat, high chair or other structured chair, carrier/sling). Reported time spent in each behaviour was then divided by 7 to obtain average time per day. Children were classified as meeting the screen time guideline if they engaged in no screen time and they were classified as meeting the restraint guidelines if parents reported 60 min or less per day for each of the five scenarios [15]. If a child met both the restraint and screen time guideline they were classified as meeting the sedentary behaviour guideline.

Parents indicated the amount of time their child spent sleeping both at night and during the day. Total sleep time was calculated by taking the sum of night and day time sleep. Children were classed as meeting the sleep guidelines if they slept between 14 and 17 h (0–3 months), 12-16 h (4–11 months) and 11-14 h (12–18 months) per day. Parents also reported the time it took for children to fall asleep during the night (night latency) and day (day latency), and the amount of time a child was awake at night.

#### Parent’s priorities

A sub sample of parents were asked about their priority areas in regards to their child’s health and development. This information was collected by asking parents to categorise the following areas from most important to least important: 1) Getting enough sleep; 2) Getting enough physical activity; 3) Getting the right amount of screen time; 4) Weaning, with a balanced diet; 5) Getting enough to eat; 6) Making sure he/she keeps a healthy weight; 7) Ensuring he/she has children to play with; 8) Attending a range of activities classes to stimulate him/her; 9) Other (define). Scores were then summarised into 3 categories, being most important (scores 1–3), average importance (scores 4–6) and least important (scores 7–9).

### Analysis

Descriptive statistics were used to summarise participant characteristics. Time spent in each of the behaviours was calculated using means and standard deviations for each of the 4 age groups (0–3.9 months; 4–7.9 months; 8–11.9 months; 12+ months). Frequencies were used to describe the proportion of children meeting the movement behaviour guidelines, the number of guidelines met and priority areas for parents. Guideline adherence was reported separately for those under 12 months of age and 12 months and older, in line with the 24-hour movement behaviour guidelines for the early years. In addition, guideline adherence was reported using 0–3.9, 4–6.9, 7–11.9 and 12+ months age categorisation to account for different developmental stages (Additional file 1). As data were not normally distributed, Mann-Whitney U-tests (continuous variables) and chi-square tests (categorical variables) were used to assess the differences between boys and girls and age groups. All analysis were conducted in SPSS version 27 (IBM Statistics) and significance was set to *p* < 0.05.

## Results

A total of 216 participants took part in the M-BAIT study; 167 (77.3%) provided valid data on all movement behaviours and were included in the current study. Participant and child characteristics are show in Table 1. Briefly, children were on average 9.6 months old (SD = 5.0 months), just over half (52.1%) of the sample were boys and 96.4% of participating parents were mothers. Participants were recruited from England (*n* = 92), Scotland (*n* = 36), and Wales (*n* = 1) (missing = 36).

**Table 1:** Participant characteristics (*n* = 167)

	% (n)	Mean	SD
**Age (mo)**		9.6	5.0
**Age groups**			
0–4	16.2 (27)		
4–8	28.7 (48)		
8–12	20.4 (34)		
12+	34.7 (58)		
**Sex (males)**	52.1 (87)		
**Country**			
England	55.1 (92)		
Scotland	21.6 (36)		
Wales	0.6 (1)		
Missing	22.8 (38)		
**Employment**			
Full time	24.0 (40)		
Part time	19.8 (33)		
Self-employed	1.8 (3)		
Maternity/paternity leave	47.9 (80)		
Unemployed	3.0 (5)		
Student	1.2 (2)		
Other	2.4 (4)		
**Childcare (yes)**	29.9 (50)		
**Relationship to child (mother)**	96.4 (161)		

### Levels of physical activity, sedentary behaviour and sleep per age group

Table 2 displays the time children spent in each of the behaviours. An upward trend across age groups was observed for all physical activity behaviours except for tummy time, which peaked at 4–7.9 months. For most physical activity behaviours, children aged 0–3.9 months spent least time and those age 8–11.9 and 12+ months spent most time in each of the physical activity behaviours. A similar pattern was identified for screen time. Time spent restrained fluctuated between the age groups, with parents of 8–11.9 month olds reporting the highest amount of time spent restrained. Aligned with guidelines, patterns were reversed for sleep, with older age groups reporting less total sleep, naps and less time spent awake at night. Few significant differences were found between boys and girls: boys showed higher levels of screen time at age 4–7.9 months and longer night latency at age 8–11.9 months; girls spent more time awake at night at age 4–7.9 months (Additional file 2).

**Table 2:** Levels of PA, SB and sleep per age group

	0–3.9 months (***n*** = 27)		4–7.9 months (***n*** = 48)		8–11.9 months (***n*** = 34)		12+ months (***n*** = 58)	
	Median	IQR	Median	IQR	Median	IQR	Median	IQR
**Physical activity (min/day)**								
With adult^a^	48.2	98.6	77.1	85.7	120.0	162.9	94.3	120.0
With child	17.1	60.0	25.7	34.3	34.3	82.5	57.9	188.6
Alone	17.1	38.6	30.3	42.9	42.9	51.4	42.9	42.9
Tummy time	8.6	25.0	15.0	34.3	10.0	42.1	0.0	8.6
Floor play	25.7	68.6	51.4	100.7	85.7	100.7	72.9	154.3
Outside	8.6	42.9	30.0	51.4	25.7	38.6	60.0	95.4
**Sedentary behaviour (min/day)**								
Restrained	94.3	115.7	124.3	113.2	145.7	156.8	128.6	90.0
Screen	2.3	8.6	4.3	17.1	6.4	19.3	10.7	33.2
**Sleep**								
Total sleep (hrs/day)	14.0	2.0	13.3	1.9	13.1	1.63	13.0	1.5
Night (7 pm-7 am; hrs/day)	10.0	2.0	11.0	1.0	10.5	1.0	11.0	1.0
Napping (7 am-7 pm; hrs/day)	4.0	3.0	2.4	1.0	2.6	1.0	2.0	1.5
Night latency (min/day)	20.0	19.8	15.0	19.8	15.0	21.0	20.0	21.0
Day latency (min/day)	10.0	10.2	15.0	10.2	10.0	8.4	10.0	6.0
Awake at night (min/day)	60.0	180.0	45.0	59.4	60.0	83.4	30.0	60.0

*IQR* Interquartile range; ^a^*n* = 20, 37, 31 and 51 for 0–3.9, 4–7.9, 8–11.9 and 12+ groups respectively

**Fig. 1:**
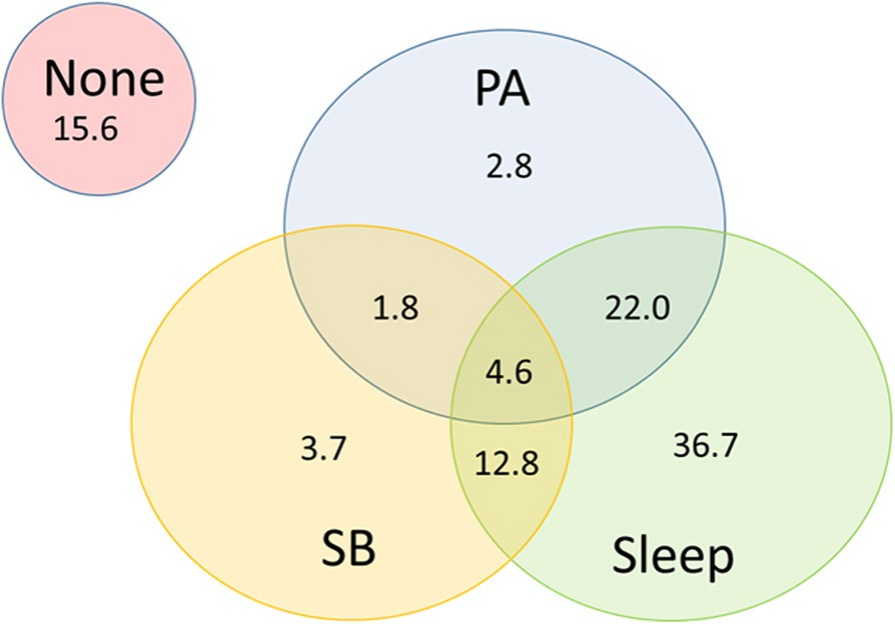
Percentage of those < 12 months meeting guidelines. Legend: PA, physical activity; SB, sedentary behaviour

Table 3 reports the proportion of children meeting each of the behaviour guidelines. For those under 12 months of age, just over 30% of children met tummy time recommendations, whereas 41.3% met the screen time guidelines. Adherence to the restraint (58.7%) and sleep guidelines (76.2%) was higher. Only 4.6% of children met all four guidelines (Fig. 1). For those 12 months and over, 24.1% met the screen time guidelines, 56.9% met the restrained guidelines and 82.8% met the sleep guidelines (Table 3). Total physical activity was not measured for those over 12 months and therefore adherence to all guidelines could not be report for this group. There was no difference between the number of boys and girls meeting guidelines across all ages (Additional file 3) or among different age group categories (Additional file 1).

**Table 3:** Proportion of children meeting the guidelines, % (n)

Individual guideline met	Total sample (***n*** = 167)	< 12 mo (***n*** = 109)	+ 12 mo (***n*** = 58)
Tummy time	NA	31.2 (34)	NA
Restraint	58.1 (97)	58.7 (64)	56.9 (33)
Screen time	35.3 (59)	41.3 (45)	24.1 (14)
Sedentary behaviour	18.6 (31)	22.9 (25)	10.3 (6)
Sleep	78.4 (131)	76.2 (83)	82.8 (48)

### Parents priorities

**Fig. 2:**
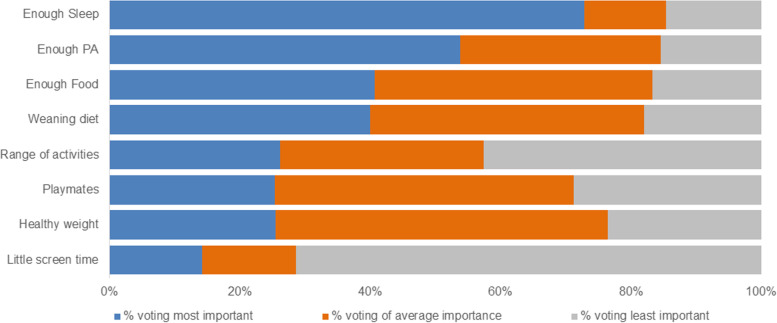
Priority topics for parents. Legend: *sleep *n* = 48, PA *n* = 52, Screen time *n* = 49, weaning *n* = 50, enough food *n* = 54, healthy weight *n* = 55, playmates *n* = 59, range of activities *n* = 61

Parents identified sleep and physical activity as top priorities for their child, with 72.9% and 53.8% respectively, listing these areas in their top 3 priorities. Limiting screen time was deemed least important, with only 14.3% listing this area as priority areas (Fig. 2).

## Discussion

This is the first study to report on the prevalence, and parental perceptions, of movement behaviours in a sample of UK infants and toddlers (0–18 months). With relatively little known about these behaviours worldwide, we note that screen time and high levels of restraint already appear to be prevalent in very young children. This suggests UK children would benefit from efforts to promote positive movement behaviours, ensuring optimal habits are formed and remain into the later preschool period.

As noted above, the number of UK infants and toddlers in this sample meeting the screen time guidelines is low, with less than half of children meeting screen, and combined sedentary behaviour, guidelines. Aligned with this, parents appeared not to be concerned about their child’s screen time, suggesting it was not a priority to limit this behaviour. The results reported in this study are slightly lower compared to those of a recent study conducted in Canada. Carson et al. reported screen time ranged from 18 min/day in those 2 months of age up to 35 min/day in those aged 6 months [11]. Previous review evidence also suggests that adherence to the screen time guideline in those under 24 months ranges from 2.3 to 83.0% [10, 16]. However, it is worth noting that the majority of the studies included in these reviews reported adherence to the screen time guidelines to be between 10 and 30%, with only 2 studies reporting adherence greater than 50%. It could be that parents use screen time as a parenting strategy, allowing them to manage their time whilst keeping children occupied. They may therefore not perceive screens to be harmful, seeing it only as a beneficial way to keep their child (ren) occupied while they complete other jobs or have necessary downtime (particularly during the pandemic). It is also possible that, given the data collection period, screens were used as a means to remain connected to family and friends during ‘lockdowns’. The nuanced reasons as to why parents allow screen time in very young children needs to be understood before interventions can be put in place.

Conversely, parents reported that a large proportion of their children were meeting sleep guidelines, which was also one of parents’ key priorities. Levels reported here were comparable to those in a Canadian cohort [11], but higher than those in Australian children [15]. It is perhaps unsurprising that parents prioritise this behaviour, given sleep (or lack thereof) often has wide-ranging influences on physical and mental health of young families, particularly during the first years of life [17]. Parents also felt that sufficient physical activity was important, but interestingly, for children in the relevant age groups few met tummy time guidelines. In line with development, tummy time increased here up to the age of 7.9 months, then declined. However, only 31% of children under 12 months meet the recommendation of 30 minutes per day in this study (0–3.9 months: 22.2%; 4–7.9 months: 33.3%; 8–11.9: 35.5%). The results of our study contradict previous studies showing higher tummy time adherence at 4 month olds. A recent Canadian study showed children aged 2 and 6 months engaged in approximately 48 and 116 min/day of tummy time respectively, substantially more than the 31 min/day reported in this study [11].

These are large differences, and may relate to differences in parental knowledge and the promotion of specific behaviours in relevant countries. For example, in Canada the guidelines for the early years were released in collaboration with ParticipACTION. ParticipACTION is a social marketing and communications organization focussed on promoting physical activity in Canada and especially successful in reaching parents [18]. It may therefore be that Canadian parents may be much more aware of the 24-hour movement behaviour guidelines compared to parents in the UK, where guideline release was not accompanied by a big marketing campaign. Whilst the inclusion of tummy time recommendations in the UK activity guidelines is a step forward, parents may not yet be aware of this and how to best go about providing tummy time safely.

### Strengths and limitations

This study was conducted in children of a range of ages, across devolved nations in the UK (i.e. England, Scotland, Wales). Using published questionnaires, previously employed in infant and toddler studies [13–15, 19], it builds on the limited evidence base for children under 2, offering important insight into movement behaviours in UK infants and toddlers. It also provides novel information about parental priorities during the early years of life, which appears to mirror how these behaviours manifest in their children. Whilst there is some evidence that tummy time is beneficial to children’s gross motor and total development, and ability to move in the first year of life, more evidence is required to show longer-term benefits of this behaviour relating to social and cognitive development [6]. It should also be born in mind that data collection for this study occurred during the COVID19 pandemic. It is possible that levels of screen time were therefore higher than they might have been normal, as digital technology was often the only means of seeing family members during this period, particularly in those over 12 months of age (those with children under 12 months old were partially exempt from social distancing restrictions). The use of non-validated questionnaires to measure sedentary behaviour and physical activity here, as none currently exist to measure these behaviours in this age group, is a limitation. However, as shown previously in older age groups, proxy-report measures can be of use to capture the proportion of children meeting movement guidelines, particularly for large-scale national surveillance, where device-based measures may be less practical. Though proxy-report measures can afford vital contextual information about movement behaviours in small children, they may be subject to recall or social-desirability biases. In contrast, device-based measures are beneficial to capture intensity and duration information about children’s behaviours, but given the challenges of assessing movement behaviours in young children, more work is needed to validate objective measures in this age group. Collaborative efforts should be encouraged to ensure consensus around these methods to prevent divergence of methods in elements such as cut points that has occurred in the older preschool measurement field.

## Conclusion

UK infants and toddlers (0–18 months) included in this sample appear to be subject to high levels of restraint, with screen time already prevalent in very young children. Relatively few infants engage in sufficient tummy time. Given the importance of these behaviours for child development and health, greater efforts are needed to support parents and care-providers to promote positive movement behaviours. Establish healthy levels of these behaviours early in life will not only benefit children’s health and wellbeing, but also ensure optimal habits are formed and retain into the later preschool period and beyond.

## Supplementary Information

**Additional file 1.** Proportion of children meeting the movement behaviour guidelines per age group.

**Additional file 2.** Levels of PA, SB and sleep per age group.

**Additional file 3.** The proportion of children meeting the movement behaviour guidelines split by sex.

## Data Availability

The dataset generated and analysed during the current study are not publicly available due ethical restrictions but are available from the corresponding author on reasonable request.

## Abbreviations

M-BAIT: Movement Behaviour Assessment in Infants and Toddlers
PA: Physical activity
SB: Sedentary behaviour
