# Supplementary material for: Movement behaviours and adherence to guidelines: perceptions of a sample of UK parents with children 0–18 months
Source: Int J Behav Nutr Phys Act. 2022 May 21;19:58. doi: 10.1186/s12966-022-01300-5 (PMC9124375; doi:10.1186/s12966-022-01300-5)
Supplement: Supplementary file 1 — Additional file 1. Proportion of children meeting the movement behaviour guidelines per age group. [file 12966_2022_1300_MOESM1_ESM.docx]

**Additional file 1**

Additional Table 1. Proportion of children meeting the guidelines based on age group, n (%)

| **Individual guideline met** | **0-3.9 months (n=27)** | **4-7.9 months (n=48)** | **8-11.9 months (n=34)** | **12+ months (n=58)** | **p-value** |
| --- | --- | --- | --- | --- | --- |
| Tummy time | 22.2 (6) | 33.3 (16) | 35.5 (12) | NA | 0.501 |
| Restraint | 59.3 (16) | 62.5 (30) | 52.9 (18) | 56.9 (33) | 0.849 |
| Screen time | 44.4 (12) | 41.7 (20) | 38.2 (13) | 24.1 (14) | 0.162 |
| Sedentary behaviour | 18.5 (5) | 29.2 (14) | 17.6 (6) | 10.3 (6) | 0.103 |
| Sleep | 59.3 (16) | 83.3 (40) | 79.4 (27) | 82.8 (48) | 0.065 |
