# Supplementary material for: Movement behaviours and adherence to guidelines: perceptions of a sample of UK parents with children 0–18 months
Source: Int J Behav Nutr Phys Act. 2022 May 21;19:58. doi: 10.1186/s12966-022-01300-5 (PMC9124375; doi:10.1186/s12966-022-01300-5)
Supplement: Supplementary file 2 — Additional file 2. Levels of PA, SB and sleep per age group. [file 12966_2022_1300_MOESM2_ESM.docx]

**Additional file 2**

Additional Table 2. Levels of PA, SB and sleep per age group

|  |  | **Boys** | | | **Girls** | | |
| --- | --- | --- | --- | --- | --- | --- | --- |
|  |  | **Median** | **IQR** | **Median** | | **IQR** |  |
| **0-3.9 months (n=27)** | **Physical activity (min/day)** |  |  |  | |  |  |
|  | With adult^#^ | 25.7 | 85.7 | 85.7 | | 96.4 |  |
|  | With child | 25.7 | 51.4 | 9.6 | | 60.0 |  |
|  | Alone | 8.6 | 25.7 | 38.6 | | 57.9 |  |
|  | Tummy time | 8.6 | 38.6 | 6.4 | | 18.2 |  |
|  | Floor play | 17.1 | 30.0 | 42.9 | | 77.1 |  |
|  | Outside | 4.3 | 25.7 | 12.9 | | 62.1 |  |
|  | **Sedentary behaviour (min/day)** |  |  |  | |  |  |
|  | Restrained | 94.3 | 94.3 | 102.1 | | 118.9 |  |
|  | Screen | 0.7 | 8.6 | 3.6 | | 8.6 |  |
|  | **Sleep (min/day)** |  |  |  | |  |  |
|  | Total sleep (hr/day) | 14.0 | 2.5 | 14.0 | | 1.7 |  |
|  | Night (7pm-7am; hr/day) | 9.8 | 3.0 | 10.5 | | 1.4 |  |
|  | Napping (7am-7pm; hr/day) | 4.0 | 5.0 | 3.5 | | 3.0 |  |
|  | Night latency | 30.0 | 45.0 | 17.5 | | 19.8 |  |
|  | Day latency | 15.0 | 19.8 | 10.0 | | 9.0 |  |
|  | Awake at night | 60.0 | 165.0 | 55.0 | | 150.0 |  |
| **4-7.9 months (n=48)** | **Physical activity (min/day)** |  |  |  | |  |  |
|  | With adult^#^ | 102.9 | 137.1 | 64.3 | | 69.6 |  |
|  | With child | 25.7 | 27.9 | 17.1 | | 55.7 |  |
|  | Alone | 38.6 | 42.9 | 21.4 | | 38.6 |  |
|  | Tummy time | 17.1 | 34.3 | 10.7 | | 34.8 |  |
|  | Floor play | 72.9 | 102.9 | 42.9 | | 72.9 |  |
|  | Outside | 30.0 | 52.5 | 30.0 | | 55.7 |  |
|  | **Sedentary behaviour (min/day)** |  |  |  | |  |  |
|  | Restrained | 111.4 | 116.4 | 132.9 | | 118.9 |  |
|  | Screen* | 12.9 | 30.0 | 0.0 | | 6.4 |  |
|  | **Sleep (min/day)** |  |  |  | |  |  |
|  | Total sleep (hr/day) | 13.3 | 2.0 | 13.0 | | 2.1 |  |
|  | Night (7pm-7am; hr/day) | 11.0 | 1.3 | 10.5 | | 2.0 |  |
|  | Napping (7am-7pm; hr/day) | 2.3 | 1.5 | 2.5 | | 1.25 |  |
|  | Night latency | 15.0 | 10.2 | 20.0 | | 20.4 |  |
|  | Day latency | 15.0 | 17.4 | 10.0 | | 11.4 |  |
|  | Awake at night* | 25.0 | 60.0 | 60.0 | | 61.2 |  |
| **8-11.9 months (n=34)** | **Physical activity (min/day)** |  |  |  | |  |  |
|  | With adult^#^ | 115.7 | 152.1 | 120.0 | | 171.4 |  |
|  | With child | 17.1 | 85.7 | 34.3 | | 98.6 |  |
|  | Alone | 42.9 | 68.6 | 42.9 | | 51.4 |  |
|  | Tummy time | 8.6 | 30.0 | 17.1 | | 55.7 |  |
|  | Floor play | 94.3 | 77.1 | 85.7 | | 162.9 |  |
|  | Outside | 21.4 | 25.7 | 25.7 | | 68.6 |  |
|  | **Sedentary behaviour (min/day)** |  |  |  | |  |  |
|  | Restrained | 135.7 | 162.9 | 154.3 | | 181.4 |  |
|  | Screen | 4.3 | 27.7 | 8.6 | | 8.6 |  |
|  | **Sleep (min/day)** |  |  |  | |  |  |
|  | Total sleep (hr/day) | 13.0 | 2.5 | 13.3 | | 1.0 |  |
|  | Night (7pm-7am; hr/day) | 10.0 | 1.0 | 11.0 | | 1.5 |  |
|  | Napping (7am-7pm; hr/day) | 3.0 | 1.5 | 2.5 | | 0.5 |  |
|  | Night latency* | 25.0 | 19.8 | 10.0 | | 19.8 |  |
|  | Day latency | 10.0 | 13.2 | 10.0 | | 4.2 |  |
|  | Awake at night | 60.0 | 100.2 | 20.0 | | 60.0 |  |
| **12+ months (n=58)** | **Physical activity (min/day)** |  |  |  | |  |  |
|  | With adult^#^ | 94.3 | 162.9 | 120.0 | | 120.0 |  |
|  | With child | 34.3 | 167.1 | 85.7 | | 180.0 |  |
|  | Alone | 42.9 | 77.1 | 42.9 | | 42.9 |  |
|  | Tummy time | 0.0 | 8.6 | 0.0 | | 1.4 |  |
|  | Floor play | 77.1 | 162.9 | 68.6 | | 137.1 |  |
|  | Outside | 42.9 | 111.4 | 60.0 | | 94.3 |  |
|  | **Sedentary behaviour (min/day)** |  |  |  | |  |  |
|  | Restrained | 133.6 | 111.4 | 128.6 | | 77.1 |  |
|  | Screen | 8.6 | 25.7 | 17.1 | | 32.9 |  |
|  | **Sleep (min/day)** |  |  |  | |  |  |
|  | Total sleep (hr/day) | 13.0 | 1.5 | 13.0 | | 1.5 |  |
|  | Night (7pm-7am; hr/day) | 11.0 | 1.0 | 11.0 | | 1.5 |  |
|  | Napping (7am-7pm; hr/day) | 2.0 | 1.25 | 2.0 | | 1.2 |  |
|  | Night latency | 20.0 | 19.8 | 15.0 | | 34.8 |  |
|  | Day latency | 10.0 | 4.8 | 15.0 | | 15.0 |  |
|  | Awake at night | 30.0 | 109.8 | 20.0 | | 60.0 |  |

*P<0.05 for comparison between boys and girls. ^#^n=20, 36, 31 and 51 for 0-3.9, 4-7.9, 8-11.9 and 12+ groups respectively.
