# Supplementary material for: Movement behaviours and adherence to guidelines: perceptions of a sample of UK parents with children 0–18 months
Source: Int J Behav Nutr Phys Act. 2022 May 21;19:58. doi: 10.1186/s12966-022-01300-5 (PMC9124375; doi:10.1186/s12966-022-01300-5)
Supplement: Supplementary file 3 — Additional file 3. The proportion of children meeting the movement behaviour guidelines split by sex. [file 12966_2022_1300_MOESM3_ESM.docx]

**Additional file 3**

Additional Table 3. Proportion of children meeting the guidelines, n (%)

| **Individual guideline met** | **Total sample** | **Boys** | **Girls** | **p-value** |
| --- | --- | --- | --- | --- |
| Tummy time (<1y)* | 34 (31.2) | 18 (30.0) | 16 (32.7) | 0.766 |
| Restrained | 97 (58.1) | 53 (60.2) | 48 (57.8) | 0.750 |
| Screen time | 59 (35.3) | 29 (33.0) | 30 (36.1) | 0.661 |
| SB combined | 31 (18.6) | 17 (19.3) | 14 (16.9) | 0.678 |
| Sleep | 131 (78.4) | 70 (79.5) | 64 (77.1) | 0.699 |
| **Number of guidelines met*** |  |  |  |  |
| 0 | 4 (3.7) | 3 (5.0) | 1 (2.0) | 0.834 |
| 1 | 27 (24.8) | 16 (26.7) | 11 (22.4) |  |
| 2 | 40 (36.7) | 22 (36.7) | 18 (36.7) |  |
| 3 | 33 (30.3) | 16 (26.7) | 17 (34.7) |  |
| All | 5 (4.6) | 3 (5.0) | 2 (4.1) |  |

*n=109, n=171 for all others.
